# Effects of aleglitazar, a balanced dual peroxisome proliferator-activated receptor α/γ agonist on glycemic and lipid parameters in a primate model of the metabolic syndrome

**DOI:** 10.1186/1475-2840-10-7

**Published:** 2011-01-20

**Authors:** Barbara C Hansen, Xenia T Tigno, Agnes Bénardeau, Markus Meyer, Elena Sebokova, Jacques Mizrahi

**Affiliations:** 1 Department of Internal Medicine and Pediatrics, University of South Florida, Tampa, FL, USA; 2 Department of Molecular Pharmacology and Physiology, University of South Florida, Tampa, FL, USA; 3 F. Hoffmann-La Roche AG, Basel, Switzerland

## Abstract

**Background:**

Glycemic control and management of dyslipidemia to reduce cardiovascular risk are major therapeutic goals in individuals with type 2 diabetes mellitus (T2DM). This study was performed to evaluate the effects of aleglitazar, a balanced dual peroxisome proliferator-activated receptor α/γ (PPARα/γ) agonist, on both lipid and glycemic parameters in obese, hypertriglyceridemic, insulin-resistant rhesus monkeys.

**Methods:**

A 135-day efficacy study was performed in six rhesus monkeys. After a 28-day baseline assessment (vehicle only), monkeys received oral aleglitazar 0.03 mg/kg per day for 42 days, followed by a 63-day washout period. Plasma levels of markers of glycemic and lipid regulation were measured at baseline, at the end of the dosing period, and at the end of the washout period.

**Results:**

Compared with baseline values, aleglitazar 0.03 mg/kg per day reduced triglyceride levels by an average of 89% (328 to 36 mg/dL; P = 0.0035 when normalized for baseline levels) and increased high-density lipoprotein cholesterol levels by 125% (46 to 102 mg/dL; P = 0.0007). Furthermore, aleglitazar reduced low-density lipoprotein cholesterol levels (41%) and increased levels of apolipoprotein A-I (17%) and A-II (17%). Aleglitazar also improved insulin sensitivity by 60% (P = 0.001). Mean body weight was reduced by 5.9% from baseline values with aleglitazar at this dose (P = 0.043).

**Conclusions:**

Aleglitazar, a dual PPARα/γ agonist, has beneficial effects on both lipid and glucose parameters and may have a therapeutic role in modifying cardiovascular risk factors and improving glycemic control in patients with T2DM.

## Introduction

According to the World Health Organization, cardiovascular disease accounts for half of all deaths in individuals with type 2 diabetes mellitus (T2DM) [1]. This highlights the need to reduce the cardiovascular risk in these individuals by management of the major T2DM-related risk factors: dyslipidemia, hyperglycemia, hypertension, and body weight [2,3]. Dyslipidemia in subjects with T2DM is characterized by elevated levels of triglycerides (TGs) and reduced levels of high-density lipoprotein cholesterol (HDL-C). Although levels of low-density lipoprotein cholesterol (LDL-C) are not significantly increased, the profile in T2DM is characterized by atherogenic small dense particles. Statin therapy has been proven to reduce effectively plasma levels of LDL-C; however, effects on HDL-C and TG levels are more modest. Therefore, a safe and efficacious therapy that can provide improvements in the overall lipid profile of patients with T2DM, while achieving good glycemic control, would have great value in reducing the risk of cardiovascular disease.

Research has shown that obesity-associated T2DM that develops spontaneously under natural and "healthy" diet conditions during adulthood in several non-human primate species, such as the rhesus monkey (*Macaca mulatta*), closely parallels the disease affecting humans [4-10]. Evidence from longitudinal studies has also provided thorough insight into the development of T2DM in rhesus monkeys [6,11]. Furthermore, the profile of naturally occurring dyslipidemia, despite a low-fat diet, closely reflects the lipid profiles of humans [12]. As such, these primates are a well-recognized, spontaneously developing animal model for the metabolic syndrome and T2DM in humans, and provide an excellent preclinical screening tool for novel antidiabetic agents [13-21].

Peroxisome proliferator-activated receptor γ (PPARγ) agonists are a class of drugs that act as insulin sensitizers, providing glycemic control. Agonists of the related α-subtype of the peroxisome proliferator-activated receptor (PPARα) improve the lipid profile, lowering levels of TGs and raising levels of HDL-C. An ideal dual PPARα/γ agonist would provide both glycemic control and an improved lipid profile at a well-tolerated therapeutic dose. Aleglitazar is a new, balanced dual PPARα/γ agonist designed to optimize glycemic and lipid benefits, and minimize PPAR-related weight gain and edema in patients with T2DM. *In vitro *binding and transactivation assays have shown that aleglitazar is a potent and high-affinity ligand for both PPARα (IC_50 _= 0.0028 μM) and PPARγ (IC_50 _= 0.0046 μM) [22]. The balanced affinity of aleglitazar for both these receptors provides a dual PPARα/γ agonist with therapeutically desirable characteristics and an improved safety profile [23], and distinguishes it from previously investigated imbalanced dual PPAR agonists that have been linked to adverse effects such as edema and renal complications [24,25].

The aim of this study was to assess the *in vivo *effects of aleglitazar on both glucose and lipid regulation in prediabetic rhesus monkeys.

## Methods

### Subjects

Six adult male rhesus monkeys with metabolic syndrome, were included in this study, based on meeting ≥3 of the following criteria: waist circumference >40 cm or waist/hip ratio >0.9, fasting plasma glucose >72 mg/dL, TG >71 mg/dL, HDL-C <60 mg/dL, and blood pressure: systolic >130 mmHg and/or diastolic >80 mmHg. This definition parallels the criteria for the diagnosis of metabolic syndrome in humans according to the Adult Treatment Panel III guidelines [26]. The animals were individually housed and cared for according to the Guide for the Care and Use of Laboratory Animals [27]. Animals had *ad libitum *access to food (monkey chow: Lab Diet 5047; Purina Mills, St Louis, MO, USA) and water, and were maintained at an ambient temperature of 20°C to 24°C with a 12-hour on-off lighting cycle. Primates were maintained in a facility approved by the Association for the Assessment and Accreditation of Laboratory and Animal Care (AAALAC) and the protocol was approved by the Institutional Animal Care and Use Committee.

### Study design

**Figure 1:**
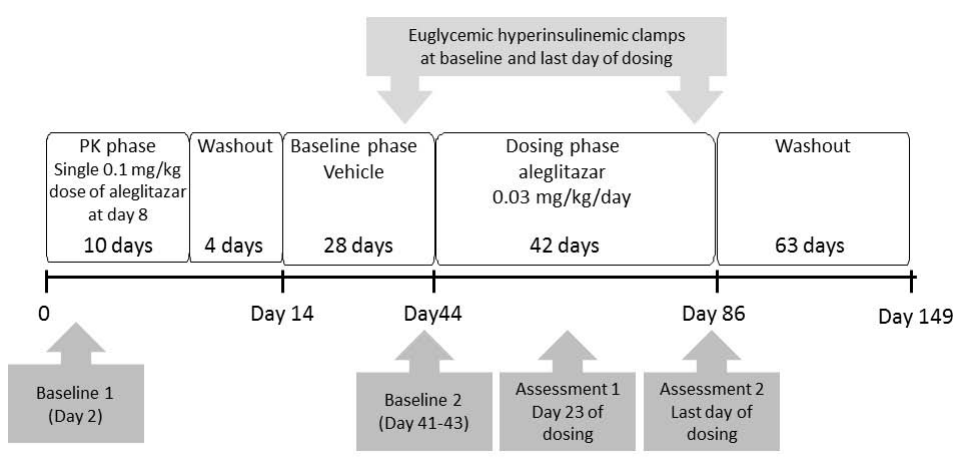
**Study design**. PK, pharmacokinetics.

Prior to initiation of the efficacy study, a 14-day pharmacokinetic (PK) assessment of a single dose of aleglitazar at 0.1 mg/kg was carried out in the same six animals; this consisted of vehicle administration (a piece of fruit ~5 g) for 7 days, followed by PK assessment of a single dose of orally administered aleglitazar (0.1 mg/kg) (results not shown), and a 4-day washout period. The efficacy study in the monkeys was initiated on day 15 and lasted for a total of 135 days. The efficacy study began on day 15 with a 28-day baseline assessment phase for all six monkeys (vehicle only). Daily dosing with aleglitazar was started on day 44 and continued at a dose of 0.03 mg/kg per day for a period of ~42 days to day ~86, a 6-week dosing period. This was followed by 63-day washout period with vehicle (fruit). A schematic overview of the study design is given in Figure 1. Following this washout period, a lower dose of aleglitazar (0.003 mg/kg per day) was assessed for a second 42-day period; however, as the majority of parameters did not return to baseline during the washout period, the results of this lower dose of aleglitazar are not presented.

In addition to standard serum chemistry and hematological assessments, blood samples were used to assess circulating levels of glucose, hemoglobin A_1c _(HbA_1c_), insulin, lipids, and adiponectin (a surrogate marker of insulin sensitivity). For the high-dose efficacy study, blood samples (2 mL each) were collected on day 2 (pre-PK baseline), day 41 to day 43 (end of vehicle baseline), after 23 days of dosing, and at the end of the 42-day dosing period. Additional blood samples were collected periodically during the washout period (14, 28, 42, 54, and 63 days after the last dose of aleglitazar).

Insulin sensitivity was determined by a euglycemic hyperinsulinemic clamp procedure performed immediately prior to the initiation of dosing with aleglitazar and immediately after completion of the 42-day dosing period, with a target clamp glucose level of 85 mg/dL and a maximum stimulated insulin level of >3000 μU/mL [4]. Electrocardiography, blood pressure, and heart rate were assessed during the pre-PK baseline, immediately prior to the initiation of aleglitazar, and after 42 days of dosing. Daily food intake (24-hour biscuit count) and biweekly body weight were recorded throughout the study.

### Assay techniques

Venous blood samples (2 mL) were drawn under light sedation (ketamine hydrochloride 10-15 mg/kg) for blood chemistry and hematology. Blood was collected for serum, and also into a vacutainer tube containing EDTA for preparation of plasma. Fasting plasma glucose (FPG) was determined using the glucose oxidase method. Fasting plasma insulin (IRI) was evaluated at LINCO Diagnostic Services (St Charles, MO, USA) using a radioimmunoassay procedure. Hematologic and blood chemistry assays were performed by Anilab, Inc (Millstone Township, NJ, USA), while the HbA_1C _level was obtained in-house using a DCA 2000+ analyzer (Bayer Diagnostics, Elkhart, IN, USA).

Total TG, HDL-C, LDL-C, and very low-density lipoprotein cholesterol (VLDL-C) were assayed by Penn Medical Laboratories (Hyattsville, MD, USA) from fresh plasma samples that had been fractionated by ultracentrifugation. In addition to the TG values as part of the basic metabolic panel, TG and lipoprotein constituents were also determined using two additional procedures to profile lipoprotein subclass distributions: (1) β-quantification (Penn Medical Laboratories) and (2) nuclear magnetic resonance (NMR) spectroscopy (LipoScience, Raleigh, NC, USA). The NMR spectroscopy lipoprotein subclass profiling technique uses the EDTA plasma from blood samples stored at -80°C [28,29] and provides a direct measure of the concentration of a specific lipoprotein subclass.

### Statistical analysis

For comparison, the baseline for the high-dose efficacy assessment was defined as the mean of the baseline values obtained at day 2 (prior to the PK study) and at days 41 to 43 (prior to dosing with aleglitazar 0.03 mg/kg), and the follow-up values were defined as the values obtained on the last day of dosing with 0.03 mg/kg aleglitazar.

Baseline and follow-up data were compared using Student's paired *t*-test. Whenever appropriate, non-parametric tests were performed. Values are expressed as mean ± standard error and a significance level of P = 0.05 was used throughout this study. The baseline and follow-up data of plasma TG concentration were normalized by expressing on a natural logarithmic scale in order to compensate for the wide and non-normally distributed plasma TG concentrations.

## Results

### Glycemic regulation

**Figure 2:**
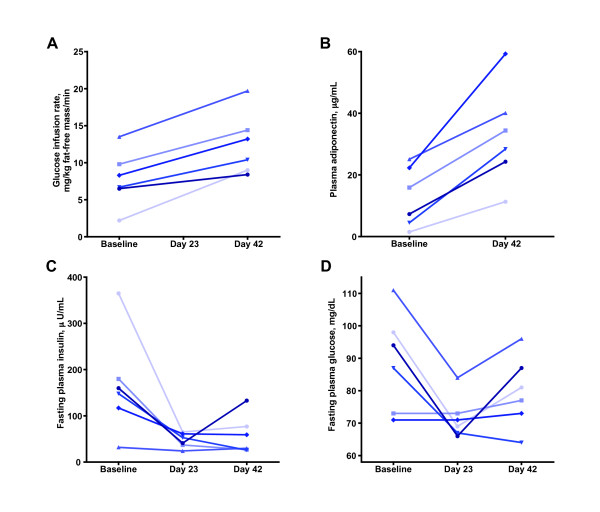
**Individual levels of A) glucose disposal rate, B) adiponectin, C) fasting plasma insulin, and D) fasting plasma glucose in monkeys given aleglitazar 0.03 mg/kg per day**. The final day of dosing at 0.03 mg/kg per day aleglitazar (labeled in the figures as day 42), due to scheduling needs, ranged across monkeys from 44 to 46 days.

Aleglitazar 0.03 mg/kg per day improved insulin sensitivity from baseline levels, as assessed by both euglycemic hyperinsulinemic clamp and adiponectin levels (a surrogate marker of insulin sensitivity). After 42 days of dosing, the mean glucose disposal rate (M-rate) increased from 7.8 to 12.5 mg/kg fat-free mass (FFM)/minute; representing a 60% increase from baseline levels (P = 0.001; Figure 2A). Similarly, the mean levels of adiponectin rose by 158% from 12.8 μg/mL at baseline to 33.0 μg/mL (P = 0.003; Figure 2B). Furthermore, circulating levels of insulin declined by 58% (146 to 61.2 μU/mL) after 42 days of dosing with aleglitazar (P = 0.042; Figure 2C).

Aleglitazar reduced FPG levels by 15% overall (89.0 to 75.3 mg/dL; P = 0.161; Figure 2D). In the subgroup of three monkeys with more elevated starting glucose levels (105.8 ± 2.0 mg/dL), the glucose-lowering effect was enhanced resulting in a 28% decline to 76.2 mg/dL. Note that normal lean rhesus monkeys have FPG levels of 60 to 75 mg/dL, about 20 mg/dL lower than those of humans. FPG levels in these three hyperglycemic monkeys did not return to hyperglycemic levels following the 63-day washout period. No episodes of hypoglycemia were observed during treatment with aleglitazar. Mean levels of HbA_1c _were non-significantly reduced from 6.7% to 5.6% after 42 days of aleglitazar treatment (P = 0.092), and remained low during the subsequent washout period.

### Lipid regulation

**Figure 3:**
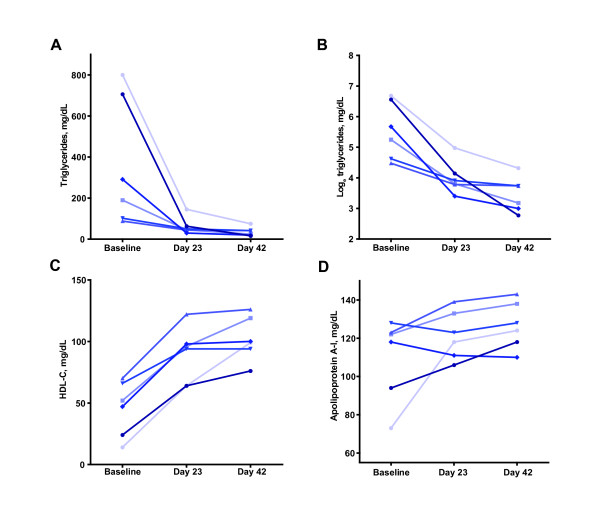
**Individual levels of a) triglyceride, b) triglyceride (normalized), c) high-density lipoprotein cholesterol, and d) apolipoprotein A-I in monkeys given aleglitazar 0.03 mg/kg per day**. The final day of dosing at 0.03 mg/kg per day aleglitazar (labeled in the figures as day 42), due to scheduling needs, ranged across monkeys from 44 to 46 days.

**Figure 4:**
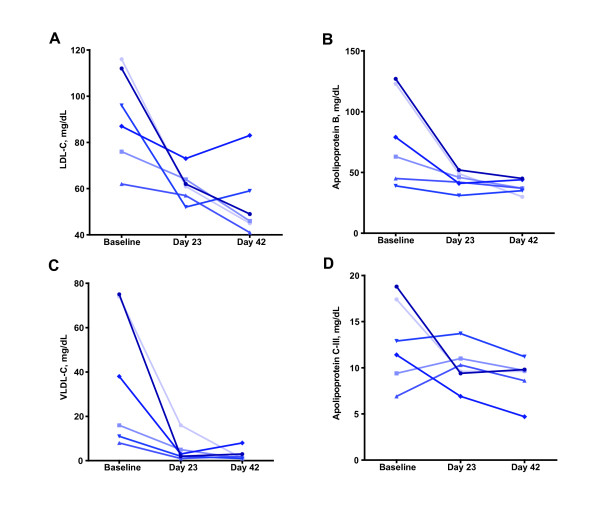
**Individual levels of A) low-density lipoprotein cholesterol, B) apolipoprotein B, C) very low-density lipoprotein cholesterol, and D) apolipoprotein C-III in monkeys given aleglitazar 0.03 mg/kg per day**. The final day of dosing at 0.03 mg/kg per day aleglitazar (labeled in the figures as day 42), due to scheduling needs, ranged across monkeys from 44 to 46 days.

Aleglitazar decreased TG levels in all monkeys by an average of 89% (328 to 36 mg/dL; P = 0.057) with 0.03 mg/kg per day (Figure 3A). Upon statistical normalization of the TG levels using logarithmic transformation, due to the wide baseline range (Figure 3A), a highly significant decrease was observed following administration of aleglitazar (Figure 3B; P = 0.0048). Total HDL-C levels increased by 125% (46 to 102 mg/dL) with aleglitazar (P = 0.0007; Figure 3C). LDL-C concentrations declined significantly by 41% with aleglitazar (92 to 54 mg/dL; P = 0.015; Figure 4A). Treatment with aleglitazar slightly increased the mean levels of apolipoprotein A-I (17% increase; P = 0.096; Figure 3D) and apolipoprotein A-II (17% increase; P = 0.111).

The NMR analysis of blood samples also revealed an 80% decrease in levels of the LDL2 (intermediate LDL - 26.6 nm diameter) fraction (P = 0.019) and a non-significant 30% increase in levels of the LDL1 (small dense LDL - 26 nm diameter) fraction (P = 0.816) following administration of aleglitazar. The mean level of the LDL3 fraction (large buoyant LDL - 27 nm diameter) was non-significantly increased by 12% (P = 0.831). Similarly, the mean level of apolipoprotein B dropped significantly by 57% (P = 0.035; Figure 4B) and levels of apolipoprotein C-III were non-significantly reduced by 36% as shown in Figure 4D (P = 0.055) for all monkeys (not normalized). Additionally, mean VLDL-C levels decreased significantly by 93% (P = 0.041; Figure 4C) and this decrease was seen for all monkeys. The largest decreases in these lipid constituents were seen in those monkeys with the highest initial baseline levels.

### Food intake and body weight

After 42 days of dosing with aleglitazar 0.03 mg/kg per day, mean body weight decreased significantly by about 5.9% from baseline (P = 0.043). A significant 32% decline in daily food intake (P = 0.0097) was observed during administration of aleglitazar.

### Safety

After 42 days of dosing with aleglitazar, mean blood urea nitrogen (BUN)/creatinine ratios increased slightly from a baseline value of 13.3 to 15.4 (P = 0.235). All biochemical and hematological parameters were, and remained, within physiologically acceptable ranges, and no evidence of peripheral edema was observed. No adverse events or episodes of hypoglycemia were observed during the study.

No changes in heart rate were observed between baseline and the end of the aleglitazar dosing period, although non-significant reductions in systolic and diastolic blood pressure (-13%; P = 0.129 and -15%; P = 0.187, respectively) were noted. Echocardiographic analysis revealed a pronounced, consistent, and significant (P = 0.0036) decrease in total peripheral resistance (which was not compensated by a reflex tachycardia), and a trend to increased cardiac output (P = 0.079) and stroke volume (P = 0.227) between baseline and the end of aleglitazar dosing. All other parameters of cardiac function that were assessed showed no relevant changes. These data are consistent with a mild hypotensive and vasodilatory effect. There was no evidence of any deleterious effect on cardiac function.

## Discussion

The results of this study in prediabetic rhesus monkeys demonstrate that the dual PPARα/γ agonist aleglitazar has marked beneficial effects on HbA_1c_, FPG, insulin sensitivity, TGs, LDL-C, HDL-C, and other lipid parameters. Laboratory and physiological assessments indicated that aleglitazar was well tolerated at 0.03 mg/kg per day.

The results indicated an overall beneficial effect of aleglitazar on cholesterol regulation in the monkeys. With aleglitazar, fasting TG and LDL-C levels were significantly reduced, while HDL-C level was significantly increased by 125%. The effects of these improvements in the lipid profile of the monkeys are difficult to extrapolate to disease progression, as a wide spectrum of lipids contributes to a varying degree to the development of cardiovascular disease or diabetes [28,30,31]. However, together with the modifications in the plasma concentrations of all classes of apolipoprotein following the administration of aleglitazar, as well as an apparent change from a profile of small dense LDL to larger LDL particles, this represents a clear shift to a less atherogenic profile in the obese monkeys and may indicate that progression of cardiovascular disease would be delayed by long-term treatment with this agonist.

In addition to the lipid benefits observed with aleglitazar, food intake and body weight were reduced during administration of the study drug. A pronounced decrease in circulating levels of TG might result from reduced food intake, and consequently, decreased body weight, as seen in animals treated with this dose of aleglitazar. This differentiates aleglitazar from PPARγ agonists that are associated with increases in body weight. The mechanism explaining these findings is not clear, but PPARα-mediated effects of the compound on adipose tissue (e.g., increased lipolysis and/or fatty acid oxidation) cannot be excluded. Phase II clinical trial data in patients with T2DM demonstrated that body weight increased to a lesser extent with aleglitazar, when compared with pioglitazone in a head-to-head comparison study [32]. Although the direct comparison between aleglitazar and pioglitazone was not performed in our study, previous thiazolidinediones studies in rhesus monkeys [15,33] support the notion that gains in body weight would be less evident with aleglitazar.

Beneficial effects of aleglitazar were also evident in improved glycemic control. Administration of aleglitazar not only reduced FPG, fasting insulin, and HbA_1c _levels in the monkeys, but also improved insulin sensitivity, as measured by the euglycemic hyperinsulinemic clamp. The 60% increase in glucose infusion rate following administration of aleglitazar was in accordance with the observed increase in plasma adiponectin, a surrogate marker of insulin sensitivity.

The observations that aleglitazar improved glucose and lipid regulation in a non-human primate model of the metabolic syndrome and impaired glucose tolerance (prediabetes), are largely consistent with previously published studies of aleglitazar in other animal models including Zucker Diabetic Fatty (ZDF) rats and human ApoAI-transgenic mice [22]. The beneficial effects of aleglitazar on the lipid and glycemic profiles of the obese monkeys in this study, especially the increase of HDL-C levels, are consistent with other dual PPAR agonists previously tested in a rhesus monkey model, such as TAK-559 [14]. Ding et al. demonstrated that TAK-559 treatment resulted in significant elevation of HDL-C, increased apolipoprotein A-I, with concomitant decreases in plasma TG and apolipoprotein B. It is believed that enhanced reverse cholesterol transport via HDL may be involved in the improved lipid profile in monkeys administered dual PPAR agonists, such as aleglitazar and TAK-559 [34]. However, unlike aleglitazar, TAK-559 did not elicit significant changes in the levels of VLDL-C or LDL-C.

Selective PPARγ agonists, such as thiazolidinediones, elicit improvements in insulin sensitivity and glucose tolerance, but are often associated with weight gain and edema in humans [35]. Selective PPARα agonists, such as fibrates, are associated with improvements in the lipid profile, but are also associated with increases in serum creatinine, with minimal effect on glycemia. Compounds that act on both receptors may represent an attractive treatment option, provided that the potential to improve both glycemic and lipid parameters can be achieved within the same therapeutic window in order to minimize the incidence of PPAR-related side effects. In this study there were no safety concerns following administration of either dose of aleglitazar, with no cases of hypoglycemia, edema, or body weight gain. The development of previous dual PPAR agonists has been halted by safety concerns; however, the safety profile of aleglitazar is encouraging [36].

The interpretation of relevance and the extrapolation of the results of the present study to human subjects must be undertaken with knowledge of its limitations that include: the subjects studied were not humans, and dose concordance between humans and monkeys is unknown.

## Conclusions

Given the close similarity between the rhesus monkey model used in this study and the diabetic state in humans, these findings suggest that aleglitazar, a dual PPARα/γ agonist, has beneficial effects on both lipid and glucose parameters and may have a therapeutic role in modifying cardiovascular risk factors and improving glycemic control in patients with T2DM.

## List of abbreviations

BUN: blood urea nitrogen; FFM: fat-free mass; FPG: fasting plasma glucose; HbA_1c_: hemoglobin A_1c_; HDL-C: high-density lipoprotein cholesterol; IRI: plasma insulin; LDL-C: low-density lipoprotein cholesterol; NMR: nuclear magnetic resonance; PK: pharmacokinetics; PPAR: peroxisome proliferator-activated receptor; T2DM: type 2 diabetes mellitus; TG: triglyceride; VLDL-C: very low-density lipoprotein cholesterol.

## Competing interests

Agnes Bénardeau, Markus Meyer, Elena Sebokova, and Jacques Mizrahi are employees of F. Hoffmann-La Roche AG. Dr. Tigno's work on this manuscript was performed during her tenure at the University of South Florida and does not reflect the view of the NIH or the United States government.

## Authors' contributions

All authors contributed to the design, analysis, and interpretation of this study. BH and XT: performed the Rhesus monkey studies. All authors contributed to the writing of this manuscript and have approved this version for submission.
